# From convention to innovation: the role of genetic modification and genome editing in Australian wheat breeding

**DOI:** 10.1093/aobpla/plaf040

**Published:** 2025-08-07

**Authors:** Oscar Carey-Fung, Alexander A T Johnson

**Affiliations:** School of BioSciences, The University of Melbourne, Parkville, VIC 3010, Australia; School of BioSciences, The University of Melbourne, Parkville, VIC 3010, Australia; Phenome, Genome & Environment

**Keywords:** agriculture, biotechnology, gene editing, GMO, OGTR

## Abstract

Wheat is the most cultivated crop worldwide, and Australia consistently ranks among the top wheat-exporting countries. Although modern technology has expanded the speed and accuracy of conventional breeding, progress is constrained by limited genetic diversity and linkage drag, with new wheat varieties often taking 8–12 years to reach the market. Biotech methods involving the transformation of foreign DNA into genomes [genetic modification (GM)], or editing of native DNA [genome editing (GEd)], provide novel opportunities to efficiently improve traits alongside conventional breeding. In 2020, the world’s first GM drought-tolerant bread wheat (HB4) hit the market in Argentina. The USA recently approved HB4 wheat for commercial cultivation, and human consumption of HB4 wheat has been approved by nine countries, including Australia. Currently, 25 countries, Australia included, have deregulated GEd crops in some form, and many other countries have indicated that they will follow suit. As of March 2025, no GM or GEd wheat is commercially grown in Australia. The rate at which private industry integrates GM and GEd into wheat breeding programmes will depend on several factors, including the regulatory consistency governing GM and GEd crops within Australia and among international trading partners, the return on investments relative to deregulation costs including licensing, the level of acceptance amongst growers and consumers, and technical considerations including wheat’s amenability to tissue culture. This review contextualizes GM and GEd applications in wheat, often drawing on examples from crop species where biotechnology has been more widely employed, and considers the key stakeholders that will shape the future of GM and GEd wheat in Australia.

## Teaser

Wheat is a cornerstone of global food security and Australia ranks among the world's leading wheat exporters. The first applications of biotechnology in wheat breeding, such as mutation breeding and marker assisted selection, aided the development of high-yielding, stress-tolerant varieties but were limited by speed and diversity of trait introgression. Genetic modification and genome editing represent more precise biotechnological tools that complement conventional breeding and enable rapid introgression of traits unimpeded by linkage drag or sexual incompatibility. Their adoption will depend on regulatory consistency, economic returns, technical feasibility, and consumer and grower acceptance. This review examines multiple factors that could shape the introduction of genetically modified and genome-edited wheat in Australia.

## History of wheat breeding in Australia

Wheat’s domestication traces back to Türkiye over 10 000 years ago, but its introduction to Australia is recent. European colonizers first brought wheat grain to Australia in 1788; however, early attempts at cultivation were hampered by Australia’s arid climate and disease pressures (State Library of New South Wales 2024). Stem Rust (caused by the fungus *Puccinia graminis*) thrived in tropical wet environments, and hot and dry climates greatly diminished yields of drought-susceptible varieties. Nevertheless, by the 1870s, Australia was frequently exporting wheat. In the 1900s, William Farrer began regularly crossing wheat varieties, representing the first example of selective wheat breeding in Australia, well before the discovery of DNA as hereditary material (Watson and Crick 1953). In 1903, Farrer released the Federation variety, which was both drought tolerant and rust resistant. Over the past century, advances in breeding, agronomy, and agricultural technology have transformed Australia’s wheat production. Reaching annual exports of ∼31.8 million metric tons in 2022–23, Australia now represents 10%–20% of the world wheat trade (Biki 2024).

## Traditional applications of biotechnology in plant breeding

Crop improvement through hybridization and artificial selection (also known as selective breeding) relies on naturally occurring genetic variation within the gene pool. The bread wheat genome (*Triticum aestivum* L.) is predicted to have 118 nucleotide substitutions and 22 insertions or deletions (indels) per plant generation (excluding variation due to transposable elements), mainly due to mistakes during DNA replication (Rafiei et al. 2024). Within a 16 billion nucleotide genome, 140 mutations represent a small source of novel variation for plant breeders to exploit. Since the 1930s, plant breeders have exposed seeds to chemicals (e.g. ethyl methanesulfonate) or irradiation (e.g. Gamma rays or X-rays) to speed up the generation of mutations across the genome in a process called mutation breeding. This process has led to the release of over 3400 registered crop varieties to date, including 275 wheat varieties (https://mvd.iaea.org). From the 1960s onwards, embryo culture, protoplast fusion, and double haploid induction using colchicine have enabled plant breeders to circumvent sexual incompatibility and perform crosses between different species (interspecific cross) or genera (intergeneric cross), further introducing novel variation (Acquaah 2015). Rapid introgression of simple genetic traits and complex polygenic traits involving many quantitative trait loci (QTLs) into an elite cultivar has been facilitated by marker-assisted selection (MAS) and genomic selection (GS). Both MAS (used for simple genetic traits) and GS (used for polygenic traits) track genetic regions associated with desirable or undesirable traits within backcrossed progeny, without the requirement for phenotyping (Collard and Mackill 2008). Additionally, double haploid induction can rapidly fix recently introgressed traits (Hassawi and Liang 1991). While MAS, GS, and double haploid induction can reduce the number of generations to introgress novel traits into an elite cultivar, speed breeding can greatly reduce generation time. The reproductive life cycle of wheat is around 4–5 months under glasshouse conditions with ambient light. However, artificial supplementation of LED lights using a 22-h photoperiod can halve the reproductive life cycle of wheat to 2–3 months (Watson et al. 2018). Still, separating genetically linked traits (linkage drag) remains a barrier when introgressing genetic traits into an elite variety.

Decreasing costs of genome sequencing over the past 20 years and advances in long-read genome sequencing (PacBio) have enabled routine QTL discovery, gene characterization, and development of genetic markers for MAS. In 2014, a draft of the bread wheat genome was published, followed by a fully annotated reference genome of cv. Chinese Spring in 2018 (Lukaszewski et al. 2014, Appels et al. 2018). In 2020, the 10+ Wheat Genomes Project provided annotated genome sequences of several wheat cultivars (Walkowiak et al. 2020). Two Australian cultivars were included within the 10+ Wheat Genomes Project: cv. Lancer [developed by Longreach) and cv. Mace (developed by Australian Grain Technologies (AGT)]. The published genomes of multiple wheat cultivars have built the foundation of a wheat pangenome and will provide an invaluable resource for precision breeding.

Hybridization between two heterotic pools of the same species to produce superior F1 offspring (heterosis) is another breeding method routinely used in maize, horticultural crops, and sometimes rice (Longin et al. 2012). Although hybrid breeding in wheat can reportedly increase yield between 3.5% and 15%, its use has yet to be widely adopted in Australia and globally (Labroo et al. 2021). Wheat is a predominately self-pollinating crop with small floral structures, making commercial-scale hybrid breeding extremely time-consuming and expensive. Gametocidal agents [also known as chemical hybridizing agents (CHAs)], nuclear male sterility (NMS), and cytoplasmic male sterility (CMS) are all methods used to prevent self-pollination by disrupting pollen development in one of the two heterotic pools. Unlike CHAs, which only temporarily disrupt pollen development, NMS and CMS require fertility restorer genes from a pollen donor for successful grain production in the F1 generation (Whitford et al. 2013, ter Steeg et al. 2022). The use of CHAs has historically been favoured over NMS and CMS systems for commercial hybrid wheat breeding; however, the timing of application and dosage can significantly impact hybrid seed production success (Gupta et al. 2019, Revell et al. 2025). To successfully deploy hybrid breeding in Australia, the costs to create heterotic pools and set up sterility and crossing systems will need to be balanced by the perceived value to growers. Additionally, a hybrid wheat system would have to operate in parallel to the current Australian End Point Royalty (EPR) scheme used to compensate breeders for commercial varieties. Under the EPR scheme, upfront seed costs are low and royalties to breeding companies are paid when grain is delivered to mills. In contrast, a hybrid wheat system would require growers to purchase new seed each year rather than resow saved seed from a previous season.

## Genetic modification applications in plant breeding

Advances in plant regeneration through cell culture have led to an explosion of modern biotechnological breeding strategies and allow the introduction of foreign DNA into plant genomes through processes like *Agrobacterium*-mediated transformation or particle bombardment (Thorpe 2007). Using these strategies, foreign DNA can be integrated into the host genome without the presence of background mutations, linkage drag, or the requirement for backcrossing in a process commonly known as genetic modification (GM). Under the broader classification of GM, cisgenesis refers to the introduction of DNA sequences in their native form from a sexually compatible species; intragenesis refers to rearranged sequences from a sexually compatible species; and transgenesis refers to native or rearranged sequences from a sexually incompatible species.

The world’s first commercialized drought-tolerant GM wheat (HB4 wheat) was developed and released in Argentina by a collaboration amongst the National Scientific and Technical Research Council, and the Institute of Agrobiotechnology of the National University of the Litoral and Bioceres Crop Solutions in 2020. The transgenic HB4 wheat contains the *HaHB4* gene from sunflower that activates the drought stress response in wheat and a *BAR* gene from *Streptomyces hygroscopicus* conferring resistance to glufosinate (herbicide tolerance) (Argentina First to Market with Drought-Resistant GM Wheat 2021, Gupta 2024). Australia, Brazil, Columbia, Indonesia, New Zealand, Nigeria, Paraguay, South Africa, and the USA have all approved HB4 for human consumption or animal feed (https://www.isaaa.org/), but commercial cultivation has only been approved in Argentina, Brazil, Paraguay, and the USA (FSANZ 2022). Field trials of HB4 wheat are underway in Australia by the wheat breeding company, Trigall, which aim to determine whether previously documented HB4 yield increases (∼6%) and greater water use efficiency (∼9%) are replicable under Australian conditions (Dealings involving Intentional Release; DIR 204) (González et al. 2019, Aslop 2024).

Canola, safflower, and cotton are the only commercially cultivated GM crops that are approved for human consumption in Australia. Oilseed crops are considered low risk in comparison to edible seeds (e.g. wheat, rice, and pulses), as their processed oils do not contain DNA. In 2021, GM canola constituted ∼26% of the total canola grown in New South Wales, Victoria, Western Australia, and South Australia (Crothers 2022). The first GM fruit in Australia—the Panama disease–resistant banana—was approved for human consumption and commercial cultivation in 2024 (FSANZ 2024). The GM banana was developed by researchers at Queensland University of Technology (QUT) who hold a license (DIR 199) for its commercial release. However, the QUT researchers state that commercial cultivation is only intended if the Panama tropical race 4 fungus threatens the banana industry in Australia (Cooper 2023). Importantly, the wide-scale adoption of GM canola, safflower, and cotton, in conjunction with recent approval for GM banana, indicates that Australia is accepting of GM crops for both food and feed purposes.

## Genome editing applications in plant breeding

Unlike random mutagenesis, genome editing (GEd) using zinc finger nucleases (ZFNs), transcription activator-like effector nucleases (TALENs), or clustered regularly interspaced short palindromic repeats (CRISPR)-associated protein 9 is site specific. Genome editing using ZFNs in plants has been used since 2005; however, CRISPR-based approaches have become the preferred GEd tool since their discovery in 2013 due to their high specificity, efficiency, modularity, and ease of delivery compared with TALENs and ZFNs (Table 1; Lloyd et al. 2005, Ran et al. 2013, Abdallah et al. 2021). The CRISPR-Cas9 system utilizes the bacterial immune system to produce a guide RNA (gRNA) and a Cas9 endonuclease *in vivo*, which together form a gRNA/Cas9 complex capable of generating site-specific double-stranded DNA breaks (Jinek et al. 2012). New transposase-based (e.g. seekRNA) and non-CRISPR re-engineered prokaryotic immune system GEd methods are proposed to offer efficiency improvements over CRISPR; however, their broad usability and performance remain to be independently validated by laboratories (Liu et al. 2024, Siddiquee et al. 2024, Faure et al. 2025).

**Table 1.** Examples of non-commercial genome editing in wheat (*T. aestivum* L.) that have produced notable phenotypes.

Phenotype	Gene target	Genome editing tool	Reference
Altered grain morphology and yield	TaIAA21	CRISPR-Cas9 (KO)	Jia et al. (2021)
	TaGASR7	CRISPR-Cas9 (KO)	Zhang et al. (2016)
	TaGW2	CRISPR-Cas9 (KO)	Zhang et al. (2016), Wang et al. (2018)
Altered flowering time, plant growth, and spike architecture	TaSPL13	CRISPR-Cas9 (miRNA site disruption)	Gupta et al. (2023)
Increased spikelet number	TaFT-D1	CRISPR-Cas9 (KO)	Chen et al. (2022)
Increased grain threshability	TaAQ and TaDq	CRISPR-Cas9 (KO)	Liu et al. (2020)
Reduced plant height	TaDEP1	CRISPR-Cas9 (KO)	Zhang et al. (2016)
Increased seed dormancy	TaQSD1	CRISPR-Cas9 (KO)	Abe et al. (2019)
Induced male sterility	TaMs1	CRISPR-Cas9 (KO)	Okada et al. (2019)
Restoration of male fertility	TaMS2	CRISPR-Cas9 (KO)	Tang et al. (2020)
Partial increase in drought tolerance	TaSAL1	CRISPR-Cas9 (KO)	Mohr et al. (2022), Abdallah et al. (2025)
Induced herbicide tolerance	TaALS-P174	deaminase–Cas9 base editing (MM)	Zhang et al. (2019)
Increased powdery mildew resistance	TaMLO	CRISPR-Cas9 (KO and CR)	Li et al. (2022), Wang et al. (2014)
	TaEDR1	CRISPR-Cas9 (KO)	Zhang et al. (2017)
Increased fusarium head blight resistance	TaHRC	CRISPR-Cas9 (KO)	Su et al. (2019)
Increased rust fungi resistance	TaPsIPK1	CRISPR-Cas9 (KO)	Wang et al. (2022)
Increased stripe rust resistance	TaCIPK14	CRISPR-Cas9 (KO)	He et al. (2023)
Increased yellow mosaic virus resistance	TaPDIL5-1	CRISPR-Cas9 (KO)	Kan et al. (2022)
Increased nitrogen use efficiency	TaARE1	CRISPR-Cas9 (KO)	Zhang et al. (2021)
Increased grain amylose content	TaSBElla	CRISPR-Cas9 (KO)	Li et al. (2021)
Increased grain micronutrient bioavailability	TaIPK1	CRISPR-Cas9 (KO)	Ibrahim et al. (2021)
Reduced grain acrylamide formation	TaASN2	CRISPR-Cas9 (KO)	Raffan et al. (2021)

Genome editing to induce gene knockouts (KO), chromosomal rearrangements (CR), or missense mutations (MM) are indicated.

Compared with GM crops which have been grown commercially since 1996, global uptake and commercial cultivation of GEd crops is still in its infancy. The first commercially released GEd crop was the high oleic soybean Calyno™ reaching the US market in 2019 and generated using TALEN. Topic Biosciences, a UK-based biotech company has developed a non-browning banana by using CRISPR-Cas9 to mutate the *polyphenol oxidase* (*PPO*) gene (also targeted by RNA interference in the Arctic® Apple by Okanagan Specialty Fruits). Tropic’s non-browning bananas have been approved for sale in the Philippines, Honduras, Columbia, the USA, and Canada and are touted to significantly combat food wastage and reduce CO_2_ emissions by 25% (https://www.isaaa.org/). At the end of 2021, Japan released the first CRISPR-Cas9 GEd crop—a tomato-enriched gamma aminobutyric acid (GABA)—which the product’s company claims to ‘reduce blood pressure’ and ‘increase relaxation’ (Waltz 2021). Although the health benefits of GABA are contentious, the release of a CRISPR-Cas9 GEd crop for enhanced human health is a milestone and may encourage the development of other nutritionally enhanced GEd crops. For bread wheat, CRISPR-Cas9 genome editing of *Asparagine Synthetase 2* (*TaASN2*) lowered asparagine biosynthesis and thereby reduced acrylamide (Group 2a carcinogen) formation (Table 1; Raffan et al. 2021). Following field trials in Europe, the GEd low acrylamide bread wheat showed no difference in yield compared with wild-type bread wheat, which brings a healthier bread wheat one step closer to consumers (Raffan et al. 2023). Powdery mildew is a fungal pathogen reported to reduce wheat yields by up to 25% (Murray and Brennan 2009). To improve powdery mildew resistance, researchers induced a 304-kb deletion in *TaMLO-B1* and knockout mutations in *TaMLO-A1* and *TaMLO-D1* in elite bread wheat cultivars using CRISPR-Cas9 (Table 1; Li et al. 2022). In 2024, China approved the release of the first GEd wheat crop, which exhibits resistance to fungus. However, whether this is the same powdery mildew–resistant mutation or resistance to another fungus is not clear (Chu 2024). Australia does not commercially cultivate any GEd wheat, and none is approved for human consumption; however, experimental field trials of GEd wheat targeting yield traits are underway by the Australian breeding company InterGrain, in collaboration with Inari, a US-based biotech company (Hobson 2024). The interest of an Australian private seed company in GEd technology indicates commercial optimism for GEd integration into the Australian wheat breeding sector.

## Transformation efficiency has until recently hindered genetic modification and genome editing applications in wheat

Transformation is an essential step in generating GM crops and is currently the preferred method of delivering CRISPR-Cas9 GEd machinery into the wheat genome for targeted mutagenesis. While delivery of CRISPR-Cas9 ribonucleoproteins (RNPs) or DNA/RNA encoding the Cas9 and gRNA sequences (transient expression) into the nucleus without genome integration has been used, low editing efficiencies have limited their widespread adoption (Zhang et al. 2016, Liang et al. 2017). The first transgenic wheat variety was successfully produced in 1992 using particle bombardment (Vasil et al. 1992). Stable transgene expression and low copy number of insertions are preferred from both a regulatory and experimental perspective. Although particle bombardment has historically been associated with high copy numbers that can lead to transgene silencing (Stam et al. 1997), preparing lower concentrations of DNA when coating gold particles can lead to higher frequencies of single-copy transgene insertions than standard methods (Ismagul et al. 2018). *Agrobacterium*-mediated transformation is an alternative to particle bombardment that generally results in a high frequency of single/low copy number of transgene insertions. *Agrobacterium*-mediated transformation relies on *Agrobacterium tumefaciens*, a tumour-inducing soil bacterium that transfers a T-DNA region from its Ti plasmid into a host plant cell with the assistance of *virulence* (*vir*) genes (Tinland 1996).

Regeneration efficiency and genotypic-specific recalcitrance to tissue culture have traditionally limited GM and GEd progress in wheat compared with soy, maize, cotton, and rice. To bypass tissue culture, the use of pollen as a vector for delivering foreign DNA into the ovule has been explored. For example, *Agrobacterium* containing a kanamycin-resistance gene was delivered into wheat spikelets, and the resulting progeny was reported to exhibit kanamycin resistance following fertilization with the transformed pollen (Hess et al. 1990). However, poor reproducibility and limited evidence of stable transgene integration have hindered adoption of the pollen pathway for wheat transformation (Langridge et al. 1992, Harwood et al. 1996). Consequentially, particle bombardment and *Agrobacterium*-mediated transformation followed by tissue culture have emerged as the preferred methods to progress GM and GEd in wheat. Immature embryos are the most commonly used explant for wheat transformation, although mature embryos, scutellum callus, shoot apical meristem, and immature inflorescences have been used with varying levels of regeneration efficiencies (Zhang et al. 2000, Patnaik et al. 2006, Supartana et al. 2006, Cui et al. 2011). Procedural factors, including varying auxin-to-cytokinin ratios, centrifuging immature wheat embryos before co-culture, and plating embryo scutellum-side up compared with scutellum-side down, can drastically impact regeneration efficiency (Kumar et al. 2017, Hayta et al. 2019, 2021, Ye et al. 2023). Wheat regeneration efficiencies using *Agrobacterium*-mediated transformation on immature embryos ranged from 0.1% to 4% in 1997 and increased to 25% by 2019 (Cheng et al. 1997, Hensel et al. 2017, Hayta et al. 2019, Kumar et al. 2019). Japan Tobacco Inc. developed a patented method of *Agrobacterium*-mediated transformation with immature wheat embryos with a regeneration efficiency of 40%–90%; however, other laboratories have not been able to reproduce the same transformation efficiencies with the available published information (Ishida et al. 2015). While new protocols have improved wheat regeneration rates, the sensitivity and fragility of donor and regenerating plants to environmental conditions make these techniques difficult to reproduce in laboratories with suboptimal growth facilities. Additionally, commercial wheat varieties are often recalcitrant to the aforementioned methods.

In 2020, the inclusion of morphogenic regulators in *Agrobacterium* T-DNA completely reshaped the efficiency of wheat transformation (Youngstrom et al. 2025). Morphogenic regulators, including Babyboom (BBM), Wuschel2 (WSH2), growth-regulating factors (GRF), Like-Aux1 (LAX), and DNA binding with one finger (DOF), are transcription factors that regulate cell-fate determination, embryogenesis, and regeneration in plants, often leading to cytokinin accumulation and auxin responses. The expression of single morphogenic genes or chimeric proteins incorporating interacting factors, such as GRF-interacting factor, has been driven by various promoters, including constitutive (e.g. pUBI), auxin-inducible (e.g. pNOS), seed-specific (e.g. pPLTP), or axillary meristem-specific (e.g. pAXIG1) promoters. These advancements have resulted in consistent wheat transformation efficiencies of 58%–100% using immature embryos and enabled transformation of previously recalcitrant wheat varieties (Debernardi et al. 2020, Wang et al. 2022, Johnson et al. 2023, Liu et al. 2023, Yu et al. 2024). Although developmental defects associated with morphogenic regulators have been reported, these pleiotropic effects may be mitigated through targeted excision using a cre-lox system or by removing the entire T-DNA following mutation induction in GEd applications (Lowe et al. 2016, 2018, Johnson et al. 2023, Lee and Wang, 2023). Within the coming years, more examples of wheat improvements using GM and GEd approaches are expected to be published, largely driven by the use of morphogenic regulators that have enabled efficient wheat transformation.

## Regulatory imbalances among trading partners have generated hesitancy for commercial adoption of genetic modification and genome editing in crops

Several incidents that have occurred during the development of GM crops highlight the trade risks associated with countries that widely use GM crops compared with countries with strict GM moratoriums. In 2013, Japan suspended wheat imports from the USA after detecting an unapproved Roundup Ready transgenic wheat variety in Oregon (Mufson 2013). Monsanto had worked on Roundup Ready wheat from 1994 but discontinued development in 2003. The resulting export losses and legal actions against Monsanto underscore the substantial financial consequences of regulatory misalignment in the global trade of GM products. A similar instance occurred in 2006, when the European Union (EU) halted rice trade with the USA after detecting traces of an unapproved herbicide-resistant transgenic rice (developed by Bayer) in exported rice shipments from the USA (ABC News 2006). Again, major losses to the US farmers led to lawsuits being filed against Bayer. Given Australia’s status as a major wheat-exporting country, its crop breeding industry is likely to exercise extreme caution to avoid repeating the missteps of the US and German companies (now Bayer AG) in commercialization of GM and GEd wheat.

In Australia, experimental and commercial growth of GM crops is regulated by the Office of the Gene Technology Regulator (OGTR). While the OGTR operates as a Commonwealth regulator, individual states and territories have sometimes imposed their own moratoriums on GM crops (Crothers 2022). Victoria ended its 4-year moratorium on GM crops in 2008. Western Australia and New South Wales repealed their moratoria in 2016 and 2021, respectively, despite both states already growing some GM crops. South Australia removed its moratorium in 2020 in all areas apart from Kangaroo Island. The Northern Territory and Queensland, in contrast, have followed Commonwealth legislation regarding where OGTR-approved GM crops can be grown. Tasmania has a moratorium on GM crops in place until 2029, and the Australian Capital Territory maintains an indefinite moratorium on commercial cultivation of GM crops.

**Figure 1.**
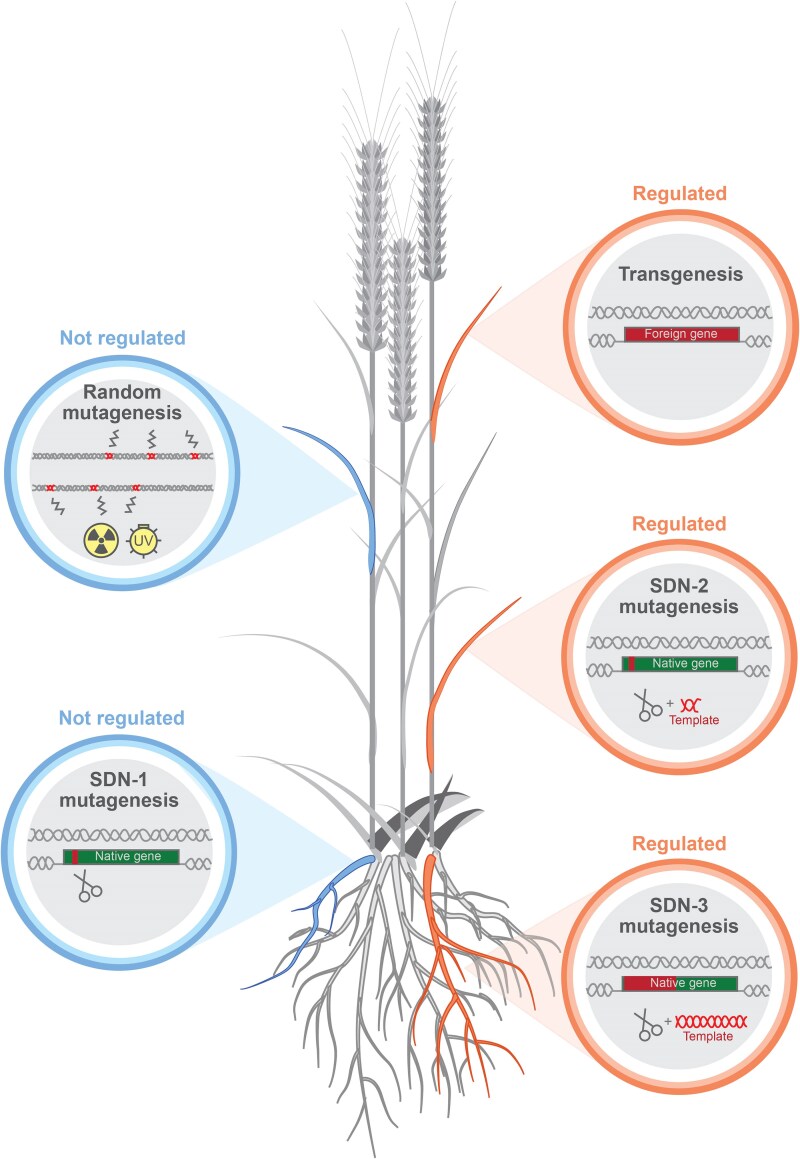
Random mutagenesis, targeted mutagenesis, and transgenesis breeding approaches and their differing regulations in Australia. Random mutagenesis and SDN-1 mutagenesis approaches are unregulated, whereas transgenesis involving the insertion of novel DNA, SDN-2 mutagenesis involving the use of a template to make small mutations, and SDN-3 mutagenesis involving the use of a template to make large insertions are regulated as GM in Australia.

Genome editing via site-directed nuclease (SDN) falls into three categories: SDN-1 edits involving double-stranded breaks repaired by error-prone non-homologous end joining that leads to small insertions or deletions (indels); SDN-2 edits utilizing a mostly homologous oligonucleotide template to introduce precise base changes via homology-directed repair; and SDN-3 edits utilizing non-homologous oligonucleotide templates to introduce new DNA sequences via homology-directed repair (Menz et al. 2020). Crops with SDN-1 mutations are not regulated by the OGTR if (1) the mutations were induced using a gRNA/Cas9 RNP, (2) the mutation was generated by a transient GEd expression cassette and that cassette has degraded or is no longer present, or (3) the mutation was generated by a stable GEd transgene, and the transgene has been removed via excision or segregated out (Fig. 1). Both SDN-2 and SDN-3 are currently regulated as GMOs under Australian law (OGTR 2018, Jones et al. 2024). The international landscape of GEd crop regulation is rapidly changing. As of March 2025, the Gene Literacy Project (https://geneticliteracyproject.org) and ISAAA (https://www.isaaa.org/) report that 25 countries have deregulated GEd crops or approved them on a case-by-case basis as of March 2025, and many countries are posed to follow (Chanikornpradit 2024). In 2024, the New Zealand government announced plans to repeal its 30-year ban on GM technology, and the EU parliament voted to ease regulation on GEd crops (Collins 2024). Although disagreements by EU member countries regarding SDN-specific legislation may delay any practical implementation, this marks a major shift in gene-technology attitudes from a continent that has historically been averse to gene technology (Menz et al. 2020). In 2023, the UK passed the Genetic Technology (Precision Breeding) Act 2023 and plans to introduce enacting legislation in 2025, which will regulate crops containing genetic changes that could have arisen naturally and through conventional breeding as non-GM—exempting SDN-1 and possibly SDN-2 mutations from GM regulation. Although concerns have been raised over potential trade issues between the UK and EU (which currently still regulates GEd as GM), the recent EU parliament vote suggests that the regulatory disharmony might be short lived. For Australia, the biggest importers of Australian wheat were China, Indonesia, the Philippines, Vietnam, and South Korea in 2023. Among these, South Korea regulates SDN-1 mutations as GM, and regulation in Vietnam is unclear, making the acceptance of GEd wheat by Asian-Pacific countries a key factor in determining GEd adoption by the Australian wheat industry (Jones et al. 2022).

## Stakeholders in Australian wheat breeding and potential attitudes towards genetic modification and genome editing of wheat

**Figure 2.**
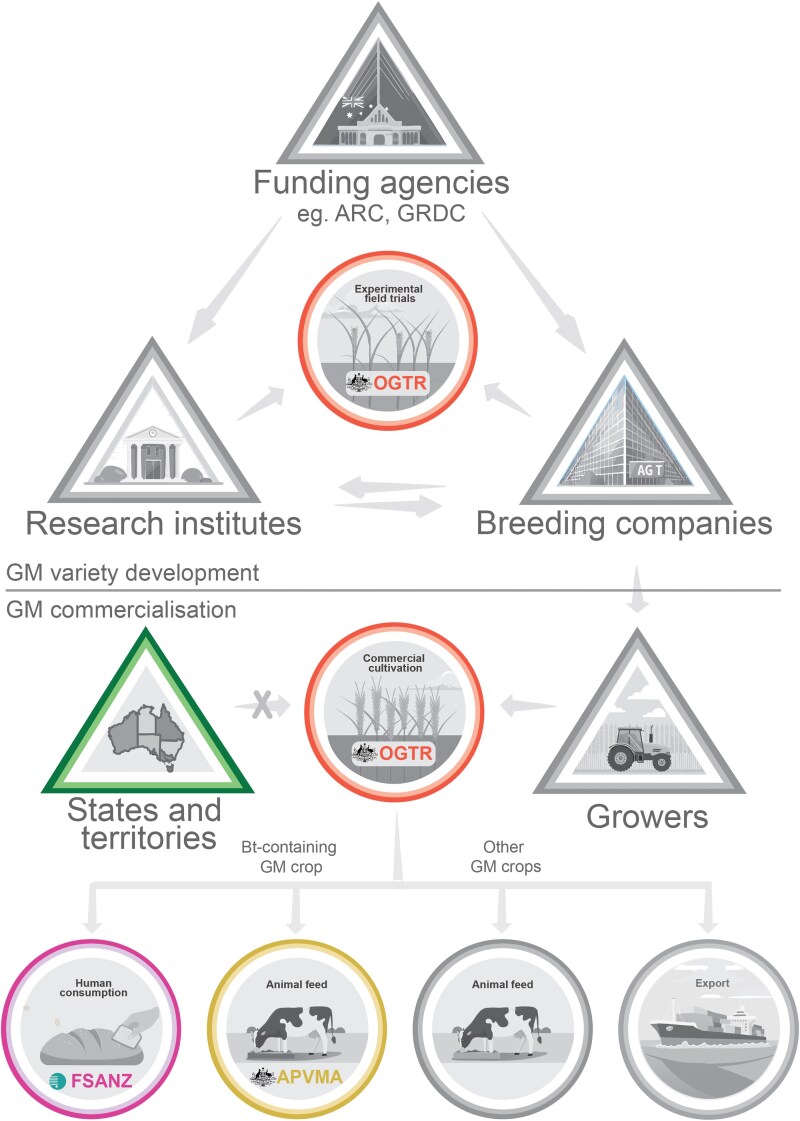
Stakeholders and regulators involved in GM development and commercialization. The stakeholders (triangles) engage in regulated (coloured) or non-regulated (grey) activities (circles) when dealing with GM crops/food/feed. The OGTR regulates commercial and experimental field trials of GM crops (red). The APVMA regulates feed for animals only if the feed contains novel pesticides (yellow). FSANZ regulates GM food for human consumption (pink). Moratoriums by some Australian states and territories (green triangle) have banned commercial cultivation of GM crops.

Prior to 1987, crop breeding in Australia was conducted primarily by universities and public research institutions, and only 5% of wheat breeders worked for private industries (Coles 2007). The introduction of the Plant Variety Rights Act (1987) and later the Plant Breeder’s Rights (PBR) Act (1994) incentivized private sector investment in plant breeding by granting exclusive commercial rights for new varieties for up to 20 years if the varieties are a product of selective breeding, new/recently exploited, distinct from other varieties, uniform, and stable throughout multiple generations. PBR laws aligned Australia’s regulatory framework with other major wheat-producing countries. Together with the EPR scheme, these changes made plant breeding more profitable and strengthened Australia’s competitiveness in the global plant breeding market by encouraging the development of wheat varieties tailored to the unique Australian climate (Sanderson and Adams 2008). Currently, most, if not all, wheat varieties are developed by private companies such as AGT (cvs. Scepter and Sunflex), LongReach (cvs. Lancer and Spitfire), and InterGrain (cvs. Vixen and Rockstar). However, universities and public institutes, including CSIRO and the Grains Research Development Corporation play key roles as industry collaborators, funders, and innovators in wheat breeding (Fig. 2). Additionally, globally funded non-for-profit research centres under the Consultative Group for International Agricultural Research, such as CIMMYT in Mexico and ICARDA in Morocco, provide valuable sources of genetic diversity and germplasm for Australian wheat breeding programmes (Trethowan et al. 2024). Breeders have estimated that 90% of domestically grown varieties in Australia have a parental lineage that traces back to CIMMYT and similarly, CIMMYT germplasm can be traced back to Australian parents (Honan 2014).

Public research institutes and universities have historically trailblazed the development of novel GM and GEd varieties in wheat, as they are not constrained by commercial licensing costs or the need for immediate return on investments (Table 1). In contrast, Australian breeding companies may be more hesitant to utilize GM and GEd technology due to the high costs of deregulating GM crops (∼$3 million AUD for a commercial license for GM safflower) and the uncertain consumer acceptance of GEd food (Jose and Prendergast 2023). Commercial use of GM or GEd crops may require breeding companies to enter complex intellectual property (IP) agreements that could limit access to germplasm. Historically, PBR protection has allowed free exchange of germplasm for breeding, contributing to genetic gains in wheat of ∼1% per year (Tadesse et al. 2019). Restricted access to GM or GEd lines may reduce the diversity of genetic material available for breeding programmes (Tadesse et al. 2019). On the other hand, GM or GEd varieties may offer breeders stronger IP protection compared with conventionally bred crops, as GM or GEd is more likely to be eligible for patents. While novel GM events are routinely patented, the patentability of GEd crops will depend on the novelty of the mutated sequence. In contrast, conventionally bred crop varieties are typically ineligible for patents due to the ‘obviousness’ of conventional breeding methodologies. Private industry may favour patent protection over PBR, as patents provide stronger strategic control over licensing and commercialization, creating greater incentive to invest in the development of GM or GEd wheat varieties.

A primary consideration for the OGTR when assessing GM crops is the ecological impact of the GM crop, including the likelihood of gene flow resulting in the unintended contamination of a non-GM field. Whilst gene flow is technically possible to non-GM fields, the associated risk is considerably lower for a primarily self-pollinated crop like wheat compared with an outcrossing species like canola, which has seen widespread GM adoption in Australia. For commercial crops used for livestock feed, regulatory approval is required from the Australian Pesticides and Veterinary Medicines Authority (APVMA) only if the GM crop produces a novel pesticide not normally found within that plant, such as Bt transgenic plants (Fig. 2; Shelton et al. 2002). Most other GM crops for livestock feed do not require registration with APVMA. For crops destined for human consumption, regulatory approval is required from the Food Standards Australia and New Zealand (FSANZ). Rigorous safety testing and public consultations are performed by FSANZ to approve a new food for human consumption, and several GM foods have now received approval (FSANZ 2017a, 2017b, 2018, 2020, 2022, 2024). Over the past 5 years, FSANZ has been reviewing their definitions of products made using New Breeding Technologies (including GEd crops) with public consultation. In June 2025, FSANZ announced a modification to their definitions of GM food (FSANZ 2025). Previously defined as ‘food produced using gene technology’, FSANZ has shifted to an updated outcome and risk-based definition for GM food. In the updated definition, GM food is defined by the presence of introduced novel DNA. This means that SND-1 GEd organisms without transgenes will be excluded from GM classification, aligning with the OGTR’s position that the risk of SDN-1 GEd is equivalent to that of conventional breeding. Australian and New Zealand Ministers are currently considering this definition change, and if approved, SDN-1 GEd products will not require FSANZ approval or GM labelling. Currently, there are no standardized guidelines for verifying that a GEd product is both transgene free and the result of an SDN-1 edit. Therefore, responsibility lies within each institution’s Institutional Biosafety Committees to ensure compliance by their researchers.

Priorities and attitudes between growers and consumers often differ for different crop traits. While growers typically favour agronomically important traits like yield, disease resistance, abiotic stress tolerance, and herbicide tolerance, consumers are likely to favour traits such as low cost, improved nutrition, extended shelf life, and reduced environmental impact. In a 2024 survey of Australian perceptions on whether GM would improve ‘our way of life’, 40% of respondence agreed and 32% disagreed (Donnelly et al. 2024). In contrast, when Australian growers were asked the same question, 63% agreed and 7% disagreed. GM or GEd wheat varieties that cater to both grower and consumer favoured traits, such as the targeting of wheat nutrition genes that enhance both grain nutrition and abiotic stress tolerance, or stacking of traits, may lead to faster commercial uptake and consumer acceptance (Kobayashi et al. 2013). However, GM crops continue to face stigma among some consumer groups. A prominent example is ‘Golden Rice’, a transgenic rice variety containing the maize *phytoene synthase* gene and a soil bacterium *phytoene desaturase* gene for the biosynthesis of β-carotene to combat human vitamin A deficiencies (Paine et al. 2005). Despite its development back in 2005, stringent regulation and anti-GM sentiment held back the commercial release of Golden Rice until 2021, when the Philippines became the first country to allow commercial cultivation (Napier et al. 2019, ISAAA 2021). Golden Rice has not been approved for commercial cultivation in Australia; however, it has been approved for human consumption by FSANZ despite objections from the ‘Action Network’ and ‘Australian Food Sovereignty Alliance’ (FSANZ 2017a). The most common reason for GM opposition is the belief that GM is ‘unnatural’ (Donnelly et al. 2024). While the distinction between GEd and GM remains poorly understood by the public, studies show that unlike other politicized scientific topics (e.g. anthropogenic climate change and vaccination) consumer education fosters a more favourable attitude towards GM and GEd foods by consumers (Donnelly et al. 2024, Bayer et al. 2025, Deka et al. 2025). Large changes in attitude trends towards GM have not been observed over the past 9 years, and in a 2024 survey, 34% of Australians found it acceptable to modify plant genes to produce food, whereas 26% of Australian found it unacceptable. Interestingly, younger Australians (16–30 years of age) were more accepting, with 42% support, compared with older people (51–75 years of age), with only 26% support (Donnelly et al. 2024). Whether a greater acceptance of GM by the same cohort of young people will remain stable as they age is unknown.

## Future perspectives

Although several Australian research groups have successfully transformed wheat (Vickers et al. 2006, Beasley et al. 2019, Regmi et al. 2020), a financially stable, open-access domestic transformation platform does not currently exist. As a result, Australian researchers often look internationally for wheat transformation. However, importation of GM wheat back into Australia is time-consuming and costly, as Australia’s strict biosecurity regulations require imported GM wheat to be grown in quarantine facilities with both Physical Containment 2 and post-entry quarantine certifications. The Commonwealth Government recently invested $20 million to set up Plant SynBio Australia (PSBA; https://bioplatforms.com/synthetic-biology/). The PSBA investment across several universities and institutes will fund infrastructure, including wheat transformation platforms. If PSBA platforms adopt an open-access fee-for-service model for public research and private industry, crucial plant breeding innovation will be supported, and the next generation of wheat scientists will be trained.

Genetic modification and GEd approaches offer powerful and complementary toolkits for Australian wheat breeders, expanding the range of traits beyond what is achievable through conventional methods. For example, linkage drag has hindered the breeding of powdery mildew resistance through conventional targeted breeding for NLRs (immune receptors imparting disease resistance), but transgenic stacking of wheat NLR-encoding genes has overcome this limitation and imparted powdery mildew resistance without pleiotropic effects (Koller et al. 2024). Similarly, while conventional breeding has failed to deliver a stable iron-biofortified wheat variety, GM approaches have achieved increases in iron by 1.5- and 1.85-fold in the grain and 2.0-fold in the white flour (Borg et al. 2012, Beasley et al. 2019, Harrington et al. 2023). Field testing of *TaASN2* knockout lines for reduced acrylamide showed a yield penalty in random mutagenesis-derived lines but not in GEd lines, highlighting the advantage of GEd compared with random mutagenesis in this case (Raffan et al. 2023). Successful integration of GM and GEd traits into Australian breeding programmes will depend on stakeholder perceptions of trait value, particularly by growers and consumers. However, plant breeding companies will ultimately determine the speed of GM and GEd uptake in wheat breeding programmes based on cost evaluations and risk assessments. The first commercially cultivated GM or GEd wheat in Australia will serve as a critical case study for breeding companies to evaluate the cost–benefit outcomes, market response and acceptance by consumers.

Whilst GM approaches have a substantial deregulatory cost and require commercial DIR licenses in Australia, SDN-1 GEd is exempt from these regulatory requirements. However, unlike conventional varieties, SDN-1 GEd products will require proof of transgene absence (e.g. whole-genome sequencing), which will incur laboratory costs. In addition, associated IP may necessitate commercial licencing. The licensing costs are likely to be determined on a case-by-case basis by the IP holders, breeding companies, and growers but would likely remain significantly lower than those for GM crops. As a result, breeders are still likely to pursue GEd as a promising approach to improve traits in wheat. Due to durum wheat’s tetraploid genome and bread wheat’s hexaploid genome, SDN-1 knockouts of single genes from a subgenome known as ‘homoeologs’ can exhibit a knock-down phenotype rather than the more deleterious knockout phenotype observed in diploid species. Random mutagenesis used in Targeting Induced Local Lesions in Genomes combined with SDN-1 GEd has already proven effective in trait improvement and would not be subject to regulatory restrictions for domestic cultivation (Uauy et al. 2017, Wang et al. 2018, Li et al. 2022). Table 1 outlines promising GEd wheat traits under development. However, some of these traits, such as *TaSAL1* knockouts for drought tolerance and *TaIPK1* knockouts for increased micronutrient bioavailability, exhibit undesirable pleiotropic effects, indicating the need for further refinement (Ibrahim et al. 2021, Abdallah et al. 2025).

Inconsistencies in SDN-1 regulation among Australia’s wheat trading partners have contributed to breeder hesitancy in adopting SDN-1 GEd within breeding programmes. While the OGTR does not regulate SDN-1 mutations, some major Australian wheat importers, such as South Korea, still regulate SDN-1 mutations as GM, raising concerns about trade compatibility. However, international momentum is building towards greater acceptance of SDN-1 technologies. In 2024, announcements made by the EU, the UK, and New Zealand to legislate or consider SDN-1 deregulation signal a formal recognition of the value that GEd can play in crop breeding. This may encourage increased investment in this technology by the Australian industry. Within Australia, the adoption of a food labelling system by FSANZ that aligns with the OGTR framework would see no compulsory labelling of SDN-1 foods. Such an approach would reflect the comparable risks of SDN-1 and conventionally bred foods in terms of food safety and may help reduce public resistance to GEd products. The shifts in domestic and international policy towards SDN-1 deregulation, along with the emergence of the first commercial GM wheat (HB4), suggest that the integration of both GM and GEd technologies in Australian wheat breeding is likely to increase over the next decade. These biotech tools will be valuable assets as the industry works to meet the demands of a growing global population and adapts to the escalating abiotic and biotic stressors driven by climate change.

## Data Availability

No new data was generated or analysed within this article.
